# Hereditary cancer syndromes: opportunities and challenges

**DOI:** 10.1186/1753-6561-7-S2-K14

**Published:** 2013-04-04

**Authors:** Patricia Ashton-Prolla

**Affiliations:** 1 Post-Graduate Program in Genetics and Molecular Biology, Federal University of Rio Grande do Sul, Brazil

## 

Prevention offers the most cost-effective long-term strategy for the control of cancer and at least one-third of all malignancies are preventable. A number of strategies have been developed over the last decades to provide opportunities for cancer prevention at different levels: primary prevention by reducing levels of exposure to high risk factors or causative agents, secondary prevention through establishment of cancer screening programs and tertiary prevention with interventions aimed at the modification of cancer outcomes, such as clinical trials and tailored therapies. In this scenario, the identification of individuals at high risk of developing certain cancers can provide an important opportunity for prevention, at different levels. The example of breast cancer will be used to further explore this concept.

Breast cancer is a significant health care problem worldwide. Among women with this diagnosis, a subset of patients with previously identifiable risk factors can be easily recognized. Two main groups of high-risk women can be described. The first is composed of women with a significantly increased lifetime risk of developing the disease when compared to the general population, and this is due to a combination of reproductive, behavioral and low-penetrance, common genetic risk factors. Several mathematical models have been developed to identify this subset of women, and specific risk-reducing interventions have been proposed (i.e. annual magnetic resonance imaging of the breast for women with estimated lifetime risks of breast cancer over 20% and chemoprevention for women with and estimated breast cancer risk greater than 1,66% in five years). A significant proportion of women, at the populational level may benefit from cancer risk assessment. In Southern Brazil, for instance, a population-based study of 9,128 women from the public primary health care system identified a lifetime breast cancer risk estimate > 20% in 8% of women in that cohort [1]. On the other hand, a study from the same region, involving 3,665 cancer-unaffected women between the ages of 40 and 69 years undergoing screening mammography, identified a significant 5-year breast cancer risk in approximately 7% [2]. Breast cancer risk estimation is important not only to direct the medical management of the patients, but has also been described as an important and independent predictor of adherence to mammographic breast cancer screening. Although the benefits of chemoprevention have been demonstrated in several large prospective clinical trials, less than one in every 10 women at high risk for breast cancer discussed her risk with a physician or was considered for risk reducing chemotherapy [3]. Thus, an effort should be undertaken not only to increase the identification of high risk patients, but also to further investigate risk reducing interventions for this group.

The second group of women at high risk for breast cancer are those who carry a germline mutation related to hereditary predisposition to the disease. Hereditary cancer syndromes underly 5-20% of all breast tumors and carriers have a significantly increased risk of developing multiple tumors at an early age [4]. The identification of hereditary cancer patients and families is essential to ensure accurate risk assessment, implementation of appropriate risk-reducing interventions and effective genetic counseling. Several hereditary breast cancer syndromes have been identified to date and mutations in many different genes have been described with these forms of the disease including those in *BRCA1*, *BRCA2*, *TP53*, *PTEN*, *ATM*, among others. The magnitude of risk associated with mutation carriage is significant, i.e. carriers of mutations in the *BRCA1* and *BRCA2* genes have an estimated lifetime risk of 70-85%, compared to the lifetime risk of women in the general population which is close to 10%. In addition, women with these mutations are at increased risk for contralateral breast tumors (40-60%) compared to those without genetic forms of the disease (5%). Taking the same example of hereditary breast and ovarian cancer syndrome due to *BRCA* mutations, several strategies for preventive interventions have been developed over the last two decades, including risk reducing surgeries, intensified and early-onset cancer screening and development of targeted therapies for tumors mainly caused by hereditary mutations. On the other hand, several challenges are still present in the diagnosis and management of hereditary breast cancer. These include our still limited knowledge of the functional impact of certain sequence alterations in known cancer predisposition genes, the absence of specific cancer predisposing mutations in certain cases (even with an obvious hereditary phenotype), the occurrence of significant phenotypic overlap between the different syndromes rendering molecular diagnosis a difficult task in certain situations and the low sensitivity and specificity of criteria currently used to define syndromes by cancer family history. Finally, there is also limited understanding of the impact of genetic modifiers and environment on the phenotype in hereditary cancer families, as well as limited knowledge on population-specific or geographically localized mutations. As example we can cite a population-specific situation related to a founder germline mutation in the tumor suppressor gene *TP53.*

The exact contribution of *TP53* germline mutations to the overall burden of cancer is still only partially known [5]. Most of the current evidence comes from studies on few families that carry highly predisposing, hotspot *TP53* mutations (TP53 mutation database). Studies in Brazil have shown that a particular mutant, p.R337H, has incomplete penetrance and may be present in a significant number of subjects due to a founder effect (estimated frequency at the populational level of 1:300 individuals in certain regions), which may not easily recognized as risk patients due to weak family history of the disease and/or later onset of tumors [6,7]. This observation raises two points: (a) the occurrence of cancer-predisposing *TP53* germline mutations may be much more common than recognized so far. This is likely the case in Southern and Southeastern Brazil for p.R337H, but may also occur in other Brazilian regions, or other parts of the world, due to similar founder mutations of incomplete penetrance; and (b) the cohort of p.R337H subjects and patients from Brazil provides a basis for both mechanistic and clinical studies.

In a recent study designed to assess the prevalence of p.R337H in breast cancer-affected women the mutation was present in breast cancer affected women with positive familial histories but no phenotypic criteria for LFS/LFL syndromes, and in a significant proportion (average among different Brazilian regions ˢ8%) of women with breast cancer and unselected for familial cancer history [8]. Mutation prevalence was significantly higher in women with breast cancer at ≤ 45 years (12·1%) than at ≥ 55 years (5·1%, p<0·001) and analysis of breast tumour tissues from mutation carriers revealed that loss of heterozygosity (LOH) was not a frequent event, different from the expected for disease-causing mutations in tumor suppressor genes. The results showed that, taking into account an estimated population prevalence of 0·3% for the Brazilian founder p.R337H allele, mutation carriers are over-represented by about 40 fold among BC patients (≤ 45 years) and by about 17 fold among late onset BC patients (≥ 55 years), suggesting that inheritance of this mutation significantly contributes to the high incidence of breast cancer observed in many parts of Brazil. This is likely the most common germline *TP53* mutation identified so far, and identification of carriers will have significant impact on design of public health interventions related to cancer control in Brazil.

## Competing interests

There are no competing interests in this presentation.
